# A new LC–MS/MS method for multiple residues/contaminants in bovine meat

**DOI:** 10.1186/s13065-021-00788-5

**Published:** 2021-12-08

**Authors:** Zehra Hajrulai-Musliu, Risto Uzunov, Stefan Jovanov, Dean Jankuloski, Velimir Stojkovski, Lazo Pendovski, James Jacob Sasanya

**Affiliations:** 1 Faculty of Veterinary Medicine-Skopje, University “Ss. Cyril and Methodius”, Lazar Pop-Trajkov 5/7, 1000 Skopje, Republic of North Macedonia; 2 International Atomic Energy Agency, Vienna International Centre, P. O. Box 100, 1400 Vienna, Austria

**Keywords:** Residues, Contaminants, LC–MS/MS, Bovine meat, Validation

## Abstract

A multi-class and multi-residue/contaminant method for the determination of veterinary drug and pesticide residues and mycotoxins in bovine meat has been developed and validated. The veterinary drug residues/contaminants included antimicrobials, anabolic hormones, lactones, β-agonists, mycotoxins, and pesticides. Isotopic labeled internal standards were included to compensate residual matrix effects. The calibrators used in the method demonstrated linearity with the R^2^ > 0.98. The decision limit (CCα) values were in the range from 0.067 to 2103.84 μg/kg, while the range for detection capability (CCβ) was from 0.083 to 2482.13 μg/kg. The limit of detection (LOD) and limit of quantification (LOQ) were in the range from 0.059 to 291.36 μg/kg, and 0.081 to 328.13 μg/kg, respectively. The recovery of analytes ranged from 61.28% to 116.20%. The intra-day coefficient of variation (CV) was from 0.97 to 25.93% and the inter-day CV was 2.30–34.04%. The method has been used for the determination of 49 residues/contaminants in bovine meat. Application of the method in routine analysis in bovine samples, revealed in limited samples the presences of enrofloxacin, oxytetracycline and sulfadiazine at the concentration of 35.22 µg/kg, 27.35 µg/kg, and 36.20 µg/kg, respectively.

**Supplementary Information:**

The online version contains supplementary material available at 10.1186/s13065-021-00788-5.

## Introduction

Important source of nutrition for many people around the world is meat. The safety of meat is seriously challenged by all kinds of low molecular weight organic contaminants, such as residues of veterinary drugs, agro-chemical residues, mycotoxins, food additives and environmental contaminants, which arouse considerable attention from people over the world [12, 14]. Liquid chromatography tandem mass spectrometry (LC–MS/MS) techniques provide a universal approach applicable to the widest number of veterinary drug residues and it is today become the technique for analysis of drug residues and contaminants in food stuffs. Until 10 years ago, many of the analytical methods mainly focused on the extraction and determination of a single class of analytes. In the monitoring programs the multi-class and multi-residue methods are increasingly being used because of their increased analytical scope and laboratory efficiency. The development of simultaneous multi-class drug residue determination is challenging task because concentrations of analyte are low in the tissues such as meat. Although the mass spectrometry is selective detection technique, the sample preparation step in analytical procedures is necessary because that reduces interference and matrix effect which occurs with the use of mass spectrometry, especially when using electrospray ionisation (ESI). Techniques such as immunoaffinity chromatography, liquid–liquid extraction, solid phase extraction (SPE) and matrix solid-phase dispersion (MSPD) are used for extraction and purification. Sometimes, for deconjugation or release of bound residues, in sample preparation the derivatization step can be incorporated [3, 13, 19, 23]. Muscle, such as other foods from animal origin, is a complex matrix. Thus, it is critical to use an efficient preparation method for sample extraction, clean-up, and concentration (when needed) before instrumental analysis. Extraction and clean-up are crucial steps in achieving the satisfactory recovery and purifying effect simultaneously for different classes of compounds from samples such as meat. However, the high concentration of proteins in meat complicates extraction and clean-up: cell compartmented enzymes (released during homogenization) can degrade some analytes during the extraction process. Proteins can also precipitate during clean-up (through solvent exchange) and cause irreversible adsorption of some analytes. Suitable sample preparation methods are therefore very critical. One such method is the QuEChERS (quick, easy, cheap, effective, rugged, and safe), originally developed for pesticide analysis [1], has also been applied to the determination of multi-class veterinary drugs in different food commodities [2, 17, 18, 20]. However, QuEChERS seems to be less suited for recovering polar veterinary drugs such as penicillins, tetracyclines and quinolones. Therefore, there is still a great need for simple and rapid multi-residue analytical methods with appropriate sample preparation techniques, for simultaneously determining veterinary drug residues, pesticides and mycotoxins in foods. Information on levels of these hazards in commonly consumed matrices is not frequently reported hence the need for development of appropriate methods to support generation of data to fill the gap. Only few methods, combining multi-detection and multi-class residues (veterinary) in a quantitative method for bovine muscle have been reported in literature. Biselli et al. [3] developed an LC–MS/MS method with a simple extraction procedure based on liquid extraction with ethylenediamine tetraacetic acid-succinate buffer and acetronitrile (MeCN). This method is suitable for determination of 84 veterinary drug residues in chicken muscle, including the following classes: benzimidazoles, quinolones, nitromidazoles, β-lactams, macrolides, triphenylmethane dyes, sulphonamides and tetracyclines. For determination of veterinary drug residues and contaminants in muscle or infant formula, in recent years, the low-temperature clean-up method has been widely developed [21, 23]. Most of the lipid components can be successfully separated from extracts with the low-temperature clean-up method. The present paper describes a sensitive and reliable LC–MS/MS including simple and generic method for the analysis of 49 veterinary drug residues and other contaminants in bovine meat.

## Materials and methods

### Reference standards

Amoxicillin (99.6%), ampicillin (99.8%), benzylpenicillin (99.3%), cloxacillin (98.7%), oxacillin (98.4%), clenbuterol HCl (99.1%), isoxsuprine HCl (100%), salbutamol (99.4%), zilpaterol HCl (96.0%), ractopamine HCl (95.5%), terbutaline hemisulfate salt (100.0%), taleranol (99.5%), 19 nortestosterone (99.8%), clostebol (99.1%), boldenone (99.1%), methyltestosterone (99.5%), testosterone (100.0%), carbofuran (99.9%), carbaryl (99.9%), parathion (99.7%), malathion (99.2%), diazinon (98.3%), dimethoate (99.8%), atrazine (99.5%), cypermethrin (98.4%), permethrin (98.1%), deltamethrin (99.9%), coumaphos (99.7%), dicholphos (99.8%), chlorpyrifos (99.8%), fenvalerate (99.4%) were purchased Sigma-Aldrich (St. Louis, MO, USA). Brombuterol (98.0%), mabuterol HCl (98.0%), cimbuterol (98.0%), clenpenterol HCl (98.0%) were obtained from Witega (Berlin, Germany). Zeranol (99.9%), stanozolol (99.8%), ceftioflur (98.0%), cephalexin (96.6%), oxytetracycline (96.5%), enrofloxacin (99.74%), ciprofloxacin (98.0%), sulfadimidine (99.6%), sulfamethoxazole (99.7%), sulfadiazine (99.8%), sulfachloropiridazine (99.1%) and sulfadimethoxine (99.7%) were obtained from Dr. Ehrenstorfer GmbH (Augsburg, Germany); ochratoxin A (≥ 98.0%) and zearalenon (99.0%) were obtained from Trylogy Analytical Laboratory, Inc. (Washington, USA).

### Isotopic labelled internal standards

Clenbuterol-d6 HCl (98.0%), brombuterol-d9 HCl (98.0%), mabuterol-d9 HCl (98.0%), clenpenterol-d5 HCl (98.0%), cimbuterol-d9 (98.0%), were obtained from Witega (Berlin, Germany); isoxsuprine-d5 hemifumarate (≥ 98.0%) and ractopamine-d6 HCl (≥ 98.0%) were obtained from the European Reference Laboratory (EURL) at RIKILT, The Netherlands, salbutamol (albuterol)-d9 (≥ 98.0%) was obtained from Dr. Ehrenstorfer GmbH (Augsburg, Germany); zilpaterol–d7 (≥ 98.0%) and β-zearalenol-d4 (≥ 98.0%) were obtained from Toronto Research Chemicals Inc. (Toronto, Canada), while terbutaline-d9 acetate hemihydrate (99.3%), flunixin-d3 (100.0%) and penicillin G-d7 N-ethylpiperidinium (98.1%) salt were obtained from Sigma-Aldrich (St. Louis, MO, USA).

### Preparation of standard solutions

Individual stock standard solutions and internal standards were prepared in methanol (MeOH) at concentration from 0.528 to 3.610 mg/mL, while the concentration of ochratoxin A was 50 μg/mL and for zearalenone was 100 μg/mL after reconstruction in methanol. After preparation of individual stock standard solutions and internal standards, the standards were divided in groups in accordance with the Maximul Recommended Residue Levels (MRL) and Minimum Required Performance Limits (MRPL) values according to the acceptance criteria of European Commision [6], European Commision [7], European Commision [8], SANCO [16], EU pesticide database [10].

The initial mixed working standards with concentration at 10 µg/mL were prepared in MeOH and kept at − 18 °C. The groups of standards were: clenbuterol, brombuterol, mabuterol in group 1; cimbuterol, clenpenterol, isoxsuprine, ractopamine in group 2; salbutamol, terbutaline, zilpaterol in group 3; testosterone, methyltestosterone, boldenone, zeranol, 19 nortestosterone, stanozolol, clostebol, taleranol in group 4; amoxicillin, ampicillin, benzylpenicillin, cloxacillin, carbaryl, parathion, dimethoate, atrazine, permethrin, dicholphos, zearalenon, ochratoxin a in group 5; enrofloxacin, ciprofloxacin, oxytetracycline, sulfadimidine, sulfamethoxazole, sulfadimethoxine, sulfadiazine, sulfachloropiridazine in group 6; carbofuran, chlorpyrifos in group 7; malathion, diazinon, coumaphos in group 8. Working standards for oxacillin, cephalexin, deltamethrin and fenvalerate were prepared at 10 µg/mL and ceftioflur and cypermethrin at 100 µg/mL.

Mixed internal standard working solutions including brombuterol-d9 HCl, cimbuterol-d9, clenbuterol-d6 HCl, clenpenterol-d5 HCl, isoxsuprine-d5 hemifumarate, mabuterol-d9 HCl, ractopamine-d6 HCl, salbutamol (albuterol)-d9, terbutaline-d9 acetate hemihydrate, zilpaterol-d7) at 100 ng/mL and (β-zearalenol-d4, flunixin-d3 and penicillin g-d7) at 10 μg/mL were prepared and kept at − 18 °C.

### Chemicals and reagents

LC–MS/MS grade MeCN, water and MeOH, HPLC grade ethylacetate (EtOAc), dichloromethane, ammonium hydroxide, n-hexane, acetic acid, ammonium acetate were obtainedfrom Carlo Erba Reagent S.A.S (Val de Reuil, France); LC–MS/MS grade formic acid was from Merck (Darmstad, Germany and Oasis HLB cartridge (500 mg/6 mL) from Waters (Milford, MA, USA).

### Sample preparation

A grand total of 100 random samples of fresh bovine meat were collected from local markets of North Macedonia. The collected samples were transported directly to the laboratory at 4 °C and then subjected to the following examination.All collected samples were homogenized. After homogenization, all samples were mixed and prepared one sample which was used for validation purposes. This process was intended to acquire appropriate-representative control sample. The control sample was checked with the analytical method to avoid pre-existing contamination with the analytes of the method’s scope. In the next step, 10 g of meat sample was fortified with the analytes and the internal standards and let to stand for 20 min. Then, 20 mL of extraction mixture (MeCN:EtOAc:acetic acid, 49.5:49.5:1, v/v/v) was added and shaken vigorously for 1 min on a vortex mixer. The mixture was then shaken for 30 min with an automated shaker and centrifuged at 8000 rpm for 10 min, at 0 °C. The extraction step was repeated and the combination of the supernatants was transferred to a 50 mL tube and kept at − 80 °C, for 20 min. The solution was filtered through filter paper (> 95% α-cellulose content, dia. 240 mm) and evaporated to nearly dryness under stream of nitrogen in a water bath at 35 °C. The extract was dissolved in 10 mL of MeOH:water (10:90, v/v) and the solution shaken for 1 min on a vortex mixer followed by cleanup by solid phase extraction using Oasis HLB cartridges. The cartridge was activated and conditioned by passing through 5 mL of MeOH and 5 mL water before supernatant was loaded and cartridges washed with 5 mL of water, and then vacuum-dried for 10 min. The residues were eluted into a test tube using 4 mL MeOH:MeCN:ammonium hydroxide (47.5:47.5:5, v/v/v) followed by 4 mL MeOH:dichloromethane (30:70, v/v). The samples were evaporated to dryness under stream of nitrogen at 35 °C and the residue reconstituted with 1 mL of MeCN:water (10:90, v/v). Defatting was attained by adding 3 mL of n-hexane of the reconstituted residues in 1 mL on MeCN:water. The solution was shaken vigorously for 1 min on a vortex mixer. After that, the lower layer was centrifuged at 10,000 rpm for 3 min and the extract was filtered through a 0.45 µm membrane filter into autosampler vials prior to LC–MS/MS analysis.

### LC–MS/MS analysis

The analysis was performed with a Waters (Milford, MA, USA) quadrupole LC–MS/MS equipped with a binary pump, vacuum degasser, thermostated autosampler and thermostated column manager. MassLynx software (Waters, Milford, MA, USA) version 4.1 was used for instrument control, data acquisition and calculation of results. Chromatographic separation was carried out using an Kinetex C18 column (50 × 2.1 mm, 2.6 μm, Phenomenex, Torrance, CA, USA).

A gradient mobile phase was used where mobile phase A consisted of 0.1% formic acid solution in water containing 5 mM ammonium acetate and mobile phase B consisted of 0.1% formic acid solution in MeCN. The elution program was as follows: 0–1 min, 95–80% A; 1–4 min, 80–60% A; 4–8 min, 60–95% A; 8–12 min, 95% A while the flow rate was 0.2 mL/min. The column temperature was 40 °C and injection volume was 10 µL.

The MS/MS acquisition was carried out using electrospray ionization positive and negative mode (ESI + and ESI −). The main MS conditions were optimized and finally set as follows: capillary voltage of 3.0 kV; source temperature of 150 °C; desolvation temperature of 400 °C; cone gas at 100 L/h; and desolvation gas at 300 L/h. Three multiple reaction monitoring (MRM) transitions of banned analytes were chosen, while for analytes with permitted limits, 2 MRM transitions were chosen. Analysis of internal standards involved one MRM transition. The optimized MRM conditions for each analyte are given in Table 1.

**Table 1:** MRM parameters for 49 compounds and 13 internal standards

Standard	Ionisation mode (ESI)	Precursor ion (m/z)	Product ion (m/z)	Collision energy (V)	Cone voltage (V)
Clenbuterol	+	276.97	**202.95** 131.87 167.77	16 30 30	22
Brombuterol	+	366.90	**292.84** 211.42 57.00	20 34 38	26
Mabuterol	+	310.95	**236.99** 216.96 57.00	18 26 30	24
Clenpenterol	+	291.00	**202.92** 131.89 167.79	16 30 28	28
Isoxsuprin	+	302.04	**164.01** 106.96 120.95	30 16 28	26
Cimbuterol	+	234.03	**159.98** 142.94 57.00	16 28 26	22
Ractopamine	+	302.04	106.96 **164.01** 120.95	28 16 24	24
Salbutamol	+	240.03	**147.96** 165.98 56.94	20 14 24	22
Zilpaterol	+	262.03	185.01 **202.05** 156.98	24 22 32	22
Terbutalin	+	226.00	**152.00** 106.97 170.00	14 30 16	26
Testosterone	+	289.16	108.99 **96.95** 289.18	24 28 28	36
Methyltestosterone	+	303.22	**96.96** 109.0 178.18	28 24 24	36
Boldenone	+	287.16	**121.03** 135.02 171.20	24 16 20	34
Zeranol	−	321.03	**90.87** 40.90 259.2	40 40 36	74
Taleranol	−	321.03	**90.87** 40.90 259.2	34 40 42	74
19 Nortestosterone	+	275.14	**109.0** 80.56 93.18	34 26 32	38
Stanozolol	+	329.22	**80.95** 95.00 121.0	46 46 42	64
Clostebol	+	323.16	**142.96** 130.98 157.13	26 26 22	40
Amoxicillin	+	367.07	**159.96** 90.89	16 40	28
Ampicillin	+	350.05	**159.94** 105.98	20 12	34
Benzylpenicillin	+	334.99	**90.96** 80.94	42 52	44
Cloxacillin	+	435.94	**159.97** 276.96	18 14	26
Oxacillin	+	402.05	**159.96** 243.03	10 12	24
Cefalexin	+	347.99	**173.93** 157.89	8 16	30
Ceftiofur	+	523.96	241.00 **125.17**	16 58	34
Enrofloxacin	+	360.05	245.09 **72.02**	30 36	36
Ciprofloxacin	+	332.01	**230.94** 245.05	40 28	38
Oxytetracycline	+	460.97	200.93 **426.02**	38 30	36
Sulfachloropyridazine	+	284.90	**155.93** 91.93	16 34	28
Sulfadiazine	+	250.97	91.93 **155.93**	30 14	28
Sulfadimetoxine	+	310.97	155.93 **91.93**	20 32	36
Sulfadimidine	+	278.95	185.93 **91.93**	18 36	34
Sulfamethoxazole	+	253.91	92.00 **155.94**	30 16	28
Carbofuran	+	222.1	**165.1** 123.0	12 22	32
Carbaryl	+	202.0	145.05 **127.0**	10 32	26
Parathion	+	308.97	148.89 **246.78**	18 14	48
Malathion	+	331.1	**127.0** 98.93	14 26	30
Diazinon	+	305.1	**169.0** 153.0	22 20	44
Dimethoate	+	229.50	**198.83** 124.84	10 20	30
Atrazine	+	216.0	**174.22** 104.14	15 30	32
Permethrin	+	390.97	**355.02** 182.92	6 12	34
Cypermethrin	+	433.0	**190.89** 90.92	20 12	28
Deltamethrin	+	229.84	**198.83** 124.85	30 14	30
Coumaphos	+	362.97	**306.86** 226.86	26 18	52
Dichlorophos	+	221.0	109.15 **127.14**	18 18	44
Chlorpyrifos	+	351.78	199.77 **296.82**	18 12	38
Fenvalerate	+	419.97	166.91 **124.88**	14 42	38
Zearalenone	−	316.97	130.87 **174.91**	30 26	62
Ochratoxin A	+	404.0	358.1 **221.0**	30 10	46
Clenbuterol-d6	+	283.03	203.56	16	22
Brombuterol-d9	+	375.93	293.87	18	24
Mabuterol-d9	+	320.07	237.94	18	24
Clenpenterol-d5	+	296.00	203.10	16	24
Isoxsuprin-d5 hemifumarat	+	308.15	168.05	16	26
Cimbuterol-d9	+	243.07	160.96	16	20
Ractopamin-d6	+	308.10	168.05	16	24
Salbutamol (Albuterol)-d9	+	249.08	148.59	20	24
Zilpaterol-d7	+	269.08	185.15	24	22
Terbutalin-d9	+	235.07	152.83	16	34
Flunixin-d3	+	300.03	263.98	36	28
Penicilin G-d7	+	455.16	114.02	10	32
β-zearalenol-d4	−	323.03	160.02	30	68

### Method validation

The analytical method was validated according to the European Commission decision 2002/657 and validation approach of the SANTE/12682/2019 guidelines [9]. For the method validation were elaluated various parameters: linearity, accuracy, precision, limit of detection (LOD), limit of quantification (LOQ), Decision limit (CCα) and Detection capability (CCβ). The linearity of the method was evaluatedon the basis of matrix-match calibration. To assess the trueness of the analytical method, recovery study was conducted, considering also that certified reference materials (CRMs) not available.

## Results and discussion

### Optimization of the MS/MS parameters

Multi-class and multi-residue LC–MS/MS analytical method, was developed for simultaneous analysis in the single run in (electrospray ionisation) ESI + and ESI −. For both ionization modes, the best sensitivity for all drugs was determined to provide the highest signals for quantification and confirmation. In positive ion mode were detected 93.55% of compounds, while in negative ionization mode were identified 6.45% of targeted compounds. The performance criteria for the analytical methods for detection of residues are prescribed in Commission Decision 2002/657/EC, therefore, this document is crucial for consultation from the laboratories for analyses of residues. According to this document, for the confirmation of banned substances (Group A) a minimum of four identification points are required, while for the confirmation of permitted substances (Group B) the minimum number of identification points is set to three. To achive maximum response for all compounds the individual standard solution with concentration from 1 μg/mL, was injected directly in the MS/MS detector. The MS/MS optimization parameters are summarized Table 1. The daughter ion with the highest intensity was used for quantification of the compounds. The quantification ion for all compounds is indicated with bold character in Table 1. The best dwell time used was between 0.01 and 0.025 s whereby good peak shapes and suitable signal to noise ratio (S/N) were attained.

### LC optimization

In order to achieve high sensitivity, good ionization, and sufficient separation with minimum interference from matrix, mobile phase and gradient are very important parameters in LC method. Different mobile phases were tested to provide the best chromatographic results, consisting of MeOH with 0.1% formic acid or MeCN with 0.1% formic acid, MeOH and MeCN (1:1) with 0.1% formic acid and water acified with formic acid (0.01% and 0.1%, v/v) and 5 mmol/L ammonium acetate. According to Ren et al. [15], Xie et al. [21] and Zhan et al. [23] formic acid or acetic acid could improve the ionization of analytes under ESI + mode, and ammonium formate or ammonium acetate could change the pH of mobile phase to improve the ionization of target compounds under ESI- mode.

In this study, the best sensitivity with better chromatographic separation and peak shape was attained using water with 0.1% formic acid and 5 mmol/L ammonium acetate as aqueous phase and MeCN with 0.1% formic acid as organic phase. Also, the peak shape and tailing were improved with addition of 5 mM ammonium acetate. Due to the differences in physiochemical characteristics of target compounds, a gradient program described in section LC–MS/MS analysis was applied in order to elute 49 compounds within 12 min.

### Optimization of the extraction procedure

The optimization of extraction methods for multi-class veterinary drugs and contaminants is challenging in part because of differences in structures and physicochemical properties; potential interfering matrices and fats/lipid and proteins in foods such as meat. Thus, the sample preparation protocol that includes simultaneous extraction of all components is an important part of a multi-residue and multi-class analysis.

Better recoveries were obtained for most of the compounds when a mixture of MeCN and EtOAc acidified with 1% acetic acid was used. The effect of sample amount (5 g and 10 g) and extraction steps on recovery was investigated (The results are given in Additional file 1: Table S1). The result showed that when 10 g of sample was extracted with twice 20 mL of MeCN:EtOAc:acetic acid (49.5:49.5:1, v/v/v), the recovery of 61.3 to 116% was achieved for a higher number of veterinary drugs, pesticides and mycotoxins (33 veterinary drugs, 14 pesticides, 2 mycotoxins). MeCN allows the precipitation of proteins and the removal of fats. However, a lot of lipid matrix also existed in extracts and to solve this problem removing the remaining fat, the low-temperature cleanup − 80 °C for 20 min was chosen. This method had been previously introduced to remove excess lipids in milk [21] and animal muscles [23] succesfully. The influence of clean-up on recoveries was also studied. In this case, three different clean up protocols were examined using DSC-MCAX (300 mg/6 mL) which contain octyl C8 and benzene sulfonic acid sorbents, Bond Elut C18 (500 mg/6 mL) which contain bonded C18 silica sorbent (octadecylsilane bonded to silica particles) and Oasis HLB (500 mg/6 mL) which contain N-vinylpyrrolidone and divinylbenzene sorbent, with analysis of spiked samples at three concentration levels and six replicates per level. The results are presented in Table 2. Results showed that for some compounds, when in the examination were used DSC-MCAX and Bond Elut cartridges, the obtained recoveries are low. The worse result for recovery obtained with DSC-MCAX cartridge was 51.45% for amoxicillin, while the lowest recovery obtained with Bond Elut cartridge was 50.12% for benzylpenicillin (Table 2). The best recoveries were obtained with Oasis HLB cartridge. For that reason, Oasis HLB cartridge with a hydrophilic–lipophilic balanced co-polymer of n-vinylpyrrolidone and divinylbenzenes, was chosen in the final method. [4], reported that the composition of these cartridges allows binding of acidic, basic, or neutral analytes. Prior to analysis with LC–MS/MS 3 mL n-hexane saturated with MeCN was added to remove the lipid material. The comparison with other methods for detection of common or similar residues and contaminants in meat are given in are given in Table 3. It should be noted that methods that can simultaneously detect veterinary drugs, pesticides and mycotoxins in meat are very rare.

**Table 2:** Recoveries per clean-up material

Analytes	Added concentration (µg/kg)	Recovery (%)		
		DSC-MCAX	Bond Elut	Oasis HLB
Clenbuterol	0.05	88.64	91.34	104.00
	0.25	82.15	77.36	105.20
	0.50	92.44	84.12	102.20
Brombuterol	0.05	71.34	80.64	75.64
	0.25	69.75	85.22	92.01
	0.50	81.32	81.34	95.34
Mabuterol	0.05	80.52	82.15	76.00
	0.25	87.24	74.22	92.00
	0.50	83.81	91.30	95.40
Clenpenterol	0.2	104.12	71.38	87.50
	0.5	101.36	78.33	93.80
	0.75	88.32	76.91	95.47
Isoxsuprin	0.2	71.26	75.12	88.50
	0.5	77.84	77.46	106.40
	0.75	70.20	80.20	102.00
Cimbuterol	0.2	81.46	77.86	95.47
	0.5	82.15	81.52	99.40
	0.75	91.46	80.16	94.53
Ractopamine	0.2	74.12	81.36	96.50
	0.5	84.56	115.32	104.40
	0.75	80.26	88.12	102.93
Salbutamol	0.5	101.46	71.36	108.00
	0.75	111.36	77.22	94.93
	1.0	100.06	74.15	86.60
Zilpaterol	0.5	77.25	80.01	81.40
	0.75	81.46	84.32	98.93
	1.0	79.32	82.56	91.10
Terbutaline	0.5	88.36	75.36	116.20
	0.75	71.32	77.88	93.20
	1.0	74.15	80.48	77.40
Testosterone	1.0	89.32	71.22	71.50
	3.0	88.36	78.36	71.66
	5.0	81.26	77.58	71.60
Boldenone	1.0	69.22	65.15	92.0
	3.0	61.14	59.36	81.34
	5.0	74.88	67.12	77.80
Clostebol	1.0	81.36	75.14	104.0
	3.0	88.32	82.88	109.34
	5.0	85.14	80.61	100.80
Methyltestosterone	1.0	85.26	74.15	80.0
	3.0	81.34	71.33	72.67
	5.0	95.16	85.14	90.20
Stanozolol	1.0	64.36	62.11	93.0
	3.0	67.36	65.66	91.33
	5.0	60.22	71.33	89.20
19 nortestosterone	1.0	79.46	81.36	105.0
	3.0	74.16	72.15	113.67
	5.0	88.12	77.48	104.20
Zeranol	1.0	81.46	70.22	75.0
	3.0	77.25	71.36	82.00
	5.0	92.18	81.35	71.80
Taleranol	1.0	75.36	80.26	71.0
	3.0	67.35	75.12	80.34
	5.0	61.36	71.36	89.60
Amoxicillin	25.0	57.46	54.36	61.89
	50.0	52.11	59.64	61.28
	75.0	51.46	55.12	79.50
Oxacillin	150.0	81.46	77.56	106.22
	300.0	74.13	71.32	92.83
	450.0	77.85	71.88	94.08
Cloxacallin	25.0	85.14	80.12	78.96
	50.0	83.22	81.36	74.94
	75.0	91.13	85.46	86.37
Benzylpenicillin	25.0	66.14	50.12	85.44
	50.0	59.23	55.14	102.56
	75.0	56.77	51.36	95.39
Ampicillin	25.0	65.17	70.64	75.64
	50.0	61.22	74.32	82.90
	75.0	69.22	75.12	96.00
Ceftioflur	500.0	88.36	81.88	95.64
	1000.0	81.23	82.56	92.32
	1500.0	84.13	90.12	93.29
Cephalexin	100.0	74.15	81.36	105.18
	200.0	76.33	90.12	106.66
	300.0	81.22	88.56	96.12
Enrofloxacin	50.0	91.36	80.92	113.78
	100.0	77.15	81.36	76.71
	150.0	81.34	90.15	82.24
Ciprofloxacin	50.0	108.12	95.16	81.13
	100.0	113.56	103.12	83.35
	150.0	104.18	97.22	92.81
Oxytetracycline	50.0	81.36	77.36	79.08
	100.0	97.32	77.18	77.25
	150.0	75.14	81.36	85.56
Sulfachloropyridazine	50.0	78.65	88.22	107.27
	100.0	77.23	90.12	82.92
	150.0	81.56	78.15	95.12
Sulfadiazine	50.0	88.15	101.32	96.98
	100.0	96.15	108.17	74.94
	150.0	92.11	97.36	90.61
Sulfadimetoxine	50.0	104.32	85.12	74.72
	100.0	92.11	91.36	85.66
	150.0	93.14	90.12	94.37
Sulfadimidine	50.0	74.15	77.36	86.30
	100.0	78.32	70.51	96.57
	150.0	77.36	72.12	99.23
Sulfamethoxazol	50.0	88.35	71.36	101.26
	100.0	71.22	77.15	84.19
	150.0	70.36	80.02	92.35
Carbofuran	5.0	71.36	84.12	79.80
	10.0	70.06	81.36	92.52
	15.0	75.18	90.12	96.93
Carbaryl	25.0	82.08	71.34	86.04
	50.0	71.46	75.18	102.20
	75.0	78.54	72.36	102.97
Parathion	25.0	59.46	61.34	65.28
	50.0	62.40	64.52	72.36
	75.0	69.77	64.36	79.82
Malathion	10.0	91.48	80.54	74.51
	20.0	98.22	84.38	86.9
	30.0	95.44	71.36	87.22
Diazinon	10.0	84.56	81.56	92.14
	20.0	78.13	77.46	99.65
	30.0	79.18	76.13	93.70
Dimethoate	25.0	67.46	70.18	65.84
	50.0	60.02	71.36	71.56
	75.0	62.15	72.15	79.80
Atrazine	25.0	74.14	104.13	85.80
	50.0	81.22	105.22	99.56
	75.0	76.54	89.36	92.85
Permethrin	25.0	92.01	91.14	104.52
	50.0	88.46	88.54	102.96
	75.0	88.18	89.13	104.17
Cypermethrin	1000.0	78.69	70.64	99.65
	2000.0	74.36	77.22	92.43
	3000.0	81.08	72.15	97.39
Deltamethrin	15.0	92.46	70.64	84.40
	30.0	103.12	70.88	92.60
	45.0	101.46	73.63	86.93
Coumaphos	10.0	90.60	91.46	84.90
	20.0	88.60	97.12	89.30
	30.0	85.12	92.15	94.43
Dichlorophos	25.0	101.35	88.36	95.36
	50.0	95.17	81.22	95.15
	75.0	97.36	82.15	97.49
Chlorpyrifos	5.0	62.08	75.22	65.6
	10.0	66.13	71.36	71.15
	15.0	65.88	70.14	77.60
Fenvalerate	12.5	69.54	64.66	61.47
	25.0	61.32	59.14	81.4
	50.0	64.12	58.36	70.36
Zearalenone	25.0	74.36	78.34	81.84
	50.0	72.15	80.12	84.36
	75.0	72.88	88.14	93.07
Ochratoxin A	25.0	81.56	65.14	73.84
	50.0	83.12	64.22	67.15
	75.0	81.48	69.77	86.85

**Table 3:** Comparison with other methods for detection of residues and contaminants in meat

Target compounds	Matrix	Sample preparation and clean up	Stationary phase	Tandem MS system	Most important differences in the method	LOQ ranges	References
84 veterinary drugs	Muscle	Liquid extraction with ethylenediamine tetraacetic acid (EDTA-succinate buffer and acetonitrile, followed by phase separation and evaporation of the supernatant	RP-LC column (pentafluoro- phenylpropyl)	MS/MS	Different extraction solvents Sample preparation without SPE extraction Different LC column Pesticides and mycotoxins are not included	2–48 µg/kg	[3]
28 Pesticides, Plant Hormone, Veterinary Drugs and Mycotoxins	Food matrices (including meat)	Liquid–liquid extraction with acidified methanol with formic acid	ZIC-pHILIC	MS/MS	Different extraction solvents Sample preparation without SPE extraction Different LC column Anabolic hormones, β-agonists, lactones, some class of antibiotics and zearalenone are not included Included only 2 pesticides	1–10 µg/kg	[5]
More than 300 veterinary drugs and pesticides	meat, fish and vegetables as baby food samples	Liquid–liquid extraction with acetonitrile containing formic acid followed by SPE extraction (Florisil cartridge)	C18 column	Orbitrap QqQ/MS	Different extraction solvents SPE extraction with different type of cartridge Different MS detector Mycotoxins are not included	10–100 µg/kg	[11]
100 veterinary drugs	Muscle	Liquid extraction with EDTA-succinate buffer and acetonitrile, followed by SPE extraction (Oasis HLB, 200 mg/6 ml)	HSS-T3	TOF	Different extraction solvents SPE extraction with different type of HLB cartridge Different LC column Different MS detector Pesticides and mycotoxins are not included	< 5 µg/kg	[12]
41 veterinary drugs	Muscle	QuEChERS (SPE extraction with Bond Elut SCX cartridge is additional step for extraction of nitroimidazoles)	C18 column	MS/MS	Different extraction solvents SPE extraction with different type of cartridge Pesticides and mycotoxins are not included	n/a*	[17]
128 Veterinary drugs, pesticides	Muscle	QuEChERS	C18 column	MS/MS	Different extraction solvents Sample preparation without SPE extraction Anabolic hormones, β-agonists, lactones, some class of antibiotics and mycotoxins are not included	n/a*	[19]
130 Veterinary drugs	Muscle	liquid–liquid extraction with acetonitrile: methanol followed by delipidation with n-hexane saturated with acetonitrile	C18 column	MS/MS	Different extraction solvents Sample preparation without SPE extraction Pesticides and mycotoxins are not included	0.1–10 µg/kg	[22]
226 veterinary drugs, pesticides, mycotoxins	Muscle	liquid–liquid extraction (EDTA–Na2, acetonitrile–ethanol, n-hexane), purification by low temperature clean-up (− 40 °C/2 h) and dispersive solid-phase extraction (D-SPE)	HSS-T3	MS/MS	Different extraction solvents Sample preparation without SPE extraction Different LC column Different temperature and time for low temperature clean-up (80 °C/20 min)	0.05–10 µg/kg	[24]

*n/a not applicable

### Validation of the method

#### Linearity

The linearity of the analytical method was determined at five differenct concentration levels for each compound. Therefore, matrix-matched calibration curve and the internal standard were utilized in the method for quantification to reduce the matrix effect. Although the method covered various different classes of veterinary pharmaceuticals, pesticides and mycotoxines the use of internal standards was only feasible for β-agonists, some antibiotics: amoxicilin, ampicilin, benzylpenicilin, oxacilin, cloxacilin, ciprofloxacin, enrofloxacinand for one mycotoxin– zearalenon (due to aspects of availability, cost, and convenience). The linear range and coefficient of corelation (R^2^) for each compound are given in Table 4. R^2^ values of most compounds was ≥ 0.990, but in 13 compounds R^2^ was below 0.99. The results correspond with the results obtained from Kaufman et al. 2008 and can probably be explained by the enzymatic/chemical instability or the extreme polarity of the compounds. The coefficient of corelation revealed good linearity in the concentration range for each compound.

**Table 4:** Linear range, Coefficient of corelation, MRL/MRPL, CCα, CCβ, LOD and LOQ

Analytes	Linear range (μg/kg)	R^2^	MRL*/MRPL** (μg/kg)	CCα (μg/kg)	CCβ (μg/kg)	LOD (μg/kg)	LOQ (μg/kg)	Internal standard
β-agonists								
Clenbuterol	0.05–0.75	0.990	0.1**	0.068	0.083	0.059	0.081	Clenbuterol-d6
Brombuterol	0.05–0.75	0.990	0.1**	0.070	0.096	0.066	0.096	Brombuterol-d9
Mabuterol	0.05–0.75	0.999	0.1**	0.067	0.091	0.059	0.100	Mabuterol-d9
Clenpenterol	0.125–0.75	0.995	0.5**	0.298	0.421	0.290	0.410	Clenpenterol-d5
Isoxsuprine	0.125–0.75	0.996	0.5**	0.313	0.422	0.281	0.440	Isosuxprin-d5 hemifumarat
Cimbuterol	0.125–0.75	0.982	0.5**	0.300	0.413	0.281	0.418	Cimbuterl-d9
Ractopamine	0.125–0.75	0.981	1.0**	0.192	0.264	0.218	0.307	Ractopamin-d6
Salbutamol	0.25–2.50	0.983	5.0**	1.083	1.398	0.546	0.732	Salbutamol-d9
Zilpaterol	0.25–2.50	0.990	5.0**	0.677	0.945	0.608	0.894	Zilpaterol-d7
Terbutalin	0.25–2.50	0.982	10.0**	0.840	1.111	0.616	0.897	Terbutalin-d9
Anabolics								
Testosteron	1.0–50.0	0.992	–	0.325	0.407	0.264	0.378	
Boldenon	1.0–50.0	0.998	1.0**	0.428	0.522	0.308	0.419	
Clostebol	1.0–50.0	0.983	–	0.742	1.000	0.597	0.782	
Methyltestosterone	1.0–50.0	0.9	1.0**	0.695	0.855	0.504	0.690	
Stanozolol	1.0–50.0	0.981	1.0**	0.548	0.745	0.498	0.672	
19 nortestosterone	1.0–50.0	0.990	1.0**	0.442	0.580	0.376	0.511	
Lactones								
Zeranol	1.0–50.0	0.990	1.0**	0.338	0.473	0.296	0.423	
Taleranol	1.0–50.0	0.999	–	0.715	0.900	0.578	0.792	
β-lactams								
Amoxicillin	25.0–200.0	0.991	50*	55.12	66.40	27.82	32.62	Penicilin G–d7
Oxacillin	50.0–500.0	0.990	300*	312.33	359.22	46.22	49.53	Penicilin G–d7
Cloxacilin	25.0–200.0	0.997	–	52.78	57.15	19.21	22.71	Penicilin G–d7
Benzylpenicillin	25.0–200.0	0.983	50*	56.32	66.43	17.21	19.46	Penicilin G–d7
Ampicillin	25.0–200.0	0.990	50*	54.12	59.31	22.31	24.36	Penicilin G–d7
Cephalosporins								
Ceftiofur	300.0–3000.0	0.991	1000*	1114.21	1272.8	291.36	328.13	
Cephalexin	50.0–500.0	0.990	200*	224.15	248.64	42.88	47.15	
Fluoroquinolones								
Enrofloxacin	25.0–200.0	0.990	100*	105.22	113.94	24.84	28.07	Flunixin d3
Ciprofloxacin	25.0–200.0	0.992	100*	108.17	132.72	25.32	29.91	Flunixin d3
Tetracyclines								
Oxytetracyclin	25.0–200.0	0.999	100*	111.45	116.74	20.78	23.48	
Sulfonamides								
Sulfachloropyridazine	25.0–200.0	0.999	100*	112.15	128.91	26.90	31.31	
Sulfadiazine	25.0–200.0	0.983	100*	108.23	117.26	27.83	32.45	
Sulfadimethoxine	25.0–200.0	0.990	100*	111.14	129.22	27.11	30.76	
Sulfadimidine	25.0–200.0	0.990	100*	104.56	109.21	26.86	30.69	
Sulfamethoxazol	25.0–200.0	0.998	100*	122.18	134.95	26.69	29.89	
OP pesticides								
Carbofuran	1.0–50.0	0.991	10*	12.26	13.81	1.08	2.12	
Carbaryl	25.0–200.0	0.990	50*	54.18	57.33	26.06	29.15	
Parathion	25.0–200.0	0.981	50*	59.12	62.78	21.36	24.35	
Malathion	1.0–50.0	0.990	20*	23.13	27.37	1.54	3.21	
Diazinon	1.0–50.0	0.982	20*	25.47	31.19	1.48	2.54	
Dimethoate	25.0–200.0	0.990	–	55.17	63.63	16.25	19.23	
Atrazine	25.0–200.0	0.981	–	54.13	60.01	18.24	20.46	
Permethrin	25.0–200.0	0.999	50*	56.38	59.06	20.51	22.18	
Cypermethrin	300.0–3000.0	0.992	2000*	2103.84	2482.13	287.26	315.22	
Deltamethrin	25.0–100.0	0.994	30*	32.18	35.08	10.14	12.78	
Coumaphos	1.0–50.0	0.991	20*	22.15	25.49	2.48	3.76	
Dichlorophos	25.0–200.0	0.990	–	56.88	61.36	19.22	22.43	
Chlorpyrifos	1.0–50.0	0.981	10*	13.56	17.08	1.63	2.44	
Fenvalerate	1.0–50.0	0.983	25*	28.12	33.46	2.28	3.17	
Mycotoxins								
Zearalenone	25.0–200.0	0.999	–	54.38	59.26	20.14	23.71	β-zearalenol-d4
Ochratoxin A	25.0–200.0	0.990	–	57.44	62.18	19.21	22.14	–

*MRL; **MRPL

*LOD, LOQ, CCα, CCβ* The decision limit (CCα) and the detection capability (CCβ) were calculated according to the 2002/657/EC requirements. The LODs were calculated from the lower concentrations of analyte i.e., the lower standards which were used for calibration curve (n = 6), and as mean value, plus 3.3 times of the calculated standard deviation and for LOQ this was plus 10 times of the calculated standard deviation. The CCα values were in the range of 0.067–2103.84 μg/kg, while the range for CCβ was from 0.083 to 2482.13 μg/kg. The LOD values were in the range of 0.059–291.36 μg/kg, while the range for LOQ was from 0.081 to 328.13 μg/kg. The gained results showed good sensitivity because the values of the LOD, LOQ, CCα and CCβ were in accordance with the requirements of 2002/657/EC. The results are show in Table 4.

#### Recovery and precision

**Fig. 1:**
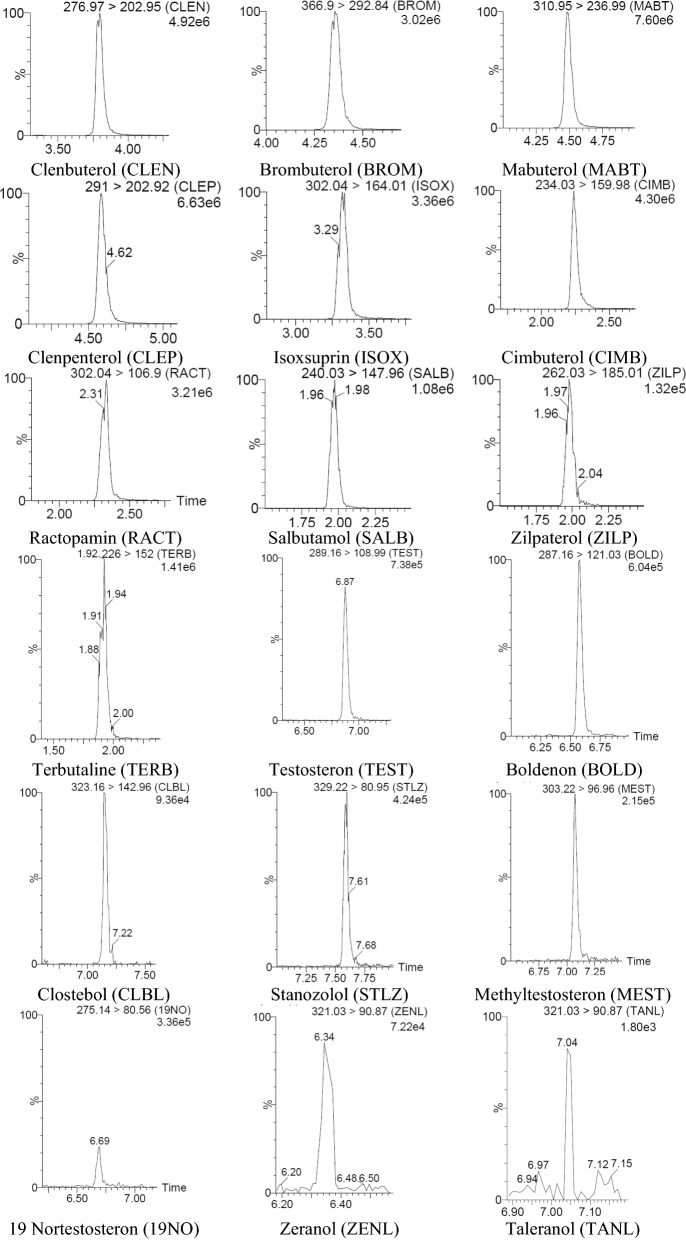

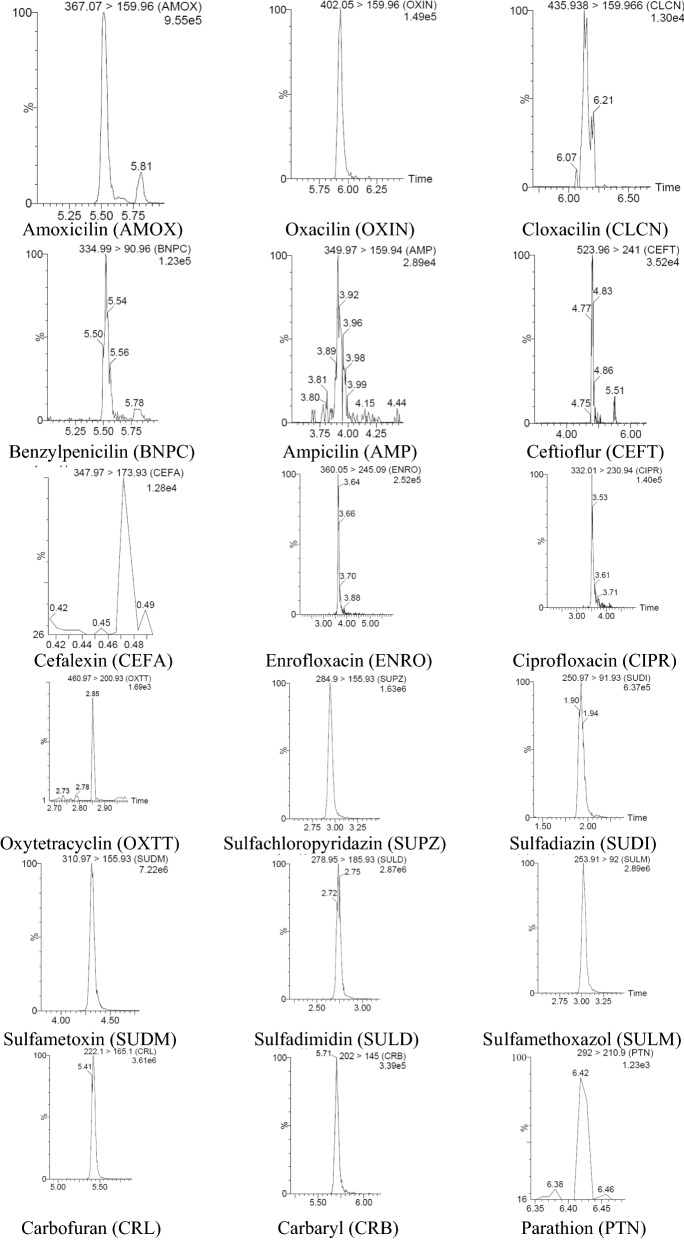

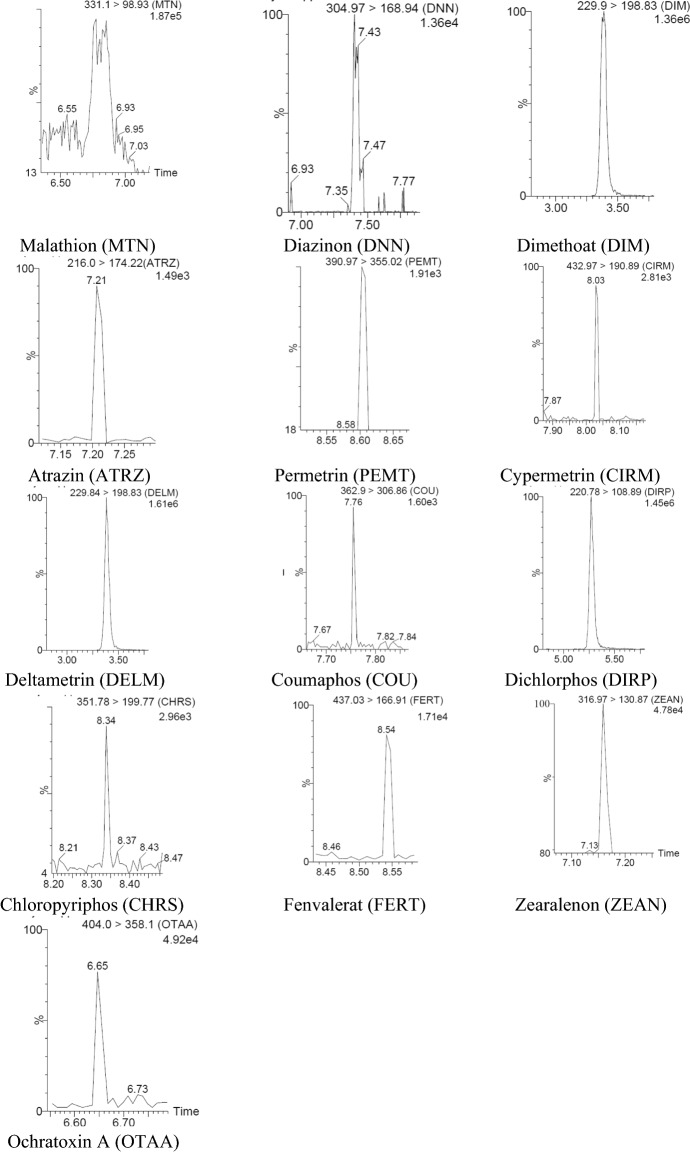
Chromatograms from spiked meat samples at the second concentration level (the second level for all analytes are given in Table 4)

Recovery and intra-day precision were evaluated by preparing spiked samples at three different concentration levels using six replicates for each concentration level in one day, while inter-day precision were evaluated by analysis of spiked samples at the same concentration levels on three consecutive days, also in six replicates for each concentration level. The spiked concentration levels and results for recovery and precion are shown in Table 5. The chromatograms from spiked meat samples and the second level are shown in Fig. 1.

**Table 5:** Recovery and precision of the method

Analytes	Added concentration (µg/kg)	Concentration in the samples (µg/kg)	Recovery (%)	Intra-day precision CV_r_ (%)	Inter-day precision CV_R_ (%)
Clenbuterol	0.05	0.052	104.00	5.54	16.49
	0.25	0.263	105.20	4.27	7.02
	0.50	0.511	102.20	14.05	7.74
Brombuterol	0.05	0.038	75.64	13.22	17.74
	0.25	0.220	92.01	4.17	12.50
	0.50	0.477	95.34	9.96	11.81
Mabuterol	0.05	0.038	76.00	4.73	10.27
	0.25	0.230	92.00	3.37	5.87
	0.50	0.477	95.40	8.23	10.37
Clenpenterol	0.2	0.175	87.50	18.78	27.23
	0.5	0.469	93.80	5.24	7.55
	0.75	0.716	95.47	1.37	9.36
Isoxsuprin	0.2	0.177	88.50	25.93	28.17
	0.5	0.532	106.40	4.51	10.67
	0.75	0.765	102.00	5.08	8.51
Cimbuterol	0.2	0.177	95.47	9.89	15.99
	0.5	0.497	99.40	10.27	14.58
	0.75	0.709	94.53	11.33	17.63
Ractopamine	0.2	0.193	96.50	6.33	11.32
	0.5	0.522	104.40	10.13	11.63
	0.75	0.772	102.93	6.57	9.69
Salbutamol	0.5	0.540	108.00	11.66	14.72
	0.75	0.712	94.93	22.13	24.83
	1.0	0.836	86.60	15.03	20.48
Zilpaterol	0.5	0.407	81.40	10.32	18.46
	0.75	0.742	98.93	8.99	16.54
	1.0	0.911	91.10	8.56	16.99
Terbutaline	0.5	0.581	116.20	1.28	5.32
	0.75	0.699	93.20	16.83	23.62
	1.0	0.774	77.40	6.10	17.09
Testosterone	1.0	0.715	71.50	8.39	13.65
	3.0	2.15	71.66	8.84	14.61
	5.0	3.58	71.60	6.70	9.26
Boldenone	1.0	0.92	92.0	5.43	9.94
	3.0	2.44	81.34	15.16	19.91
	5.0	3.89	77.80	3.89	7.50
Clostebol	1.0	1.04	104.0	12.50	15.6
	3.0	3.28	109.34	8.54	18.84
	5.0	5.04	100.80	4.17	6.70
Methyltestosterone	1.0	0.80	80.0	5.00	8.34
	3.0	2.18	72.67	9.64	14.44
	5.0	4.51	90.20	15.75	18.51
Stanozolol	1.0	0.93	93.0	5.38	9.39
	3.0	2.74	91.33	3.29	7.02
	5.0	4.46	89.20	11.44	15.09
19 nortestosterone	1.0	1.05	105.0	8.57	13.31
	3.0	3.41	113.67	6.16	10.66
	5.0	5.21	104.20	9.98	19.42
Zeranol	1.0	0.75	75.0	9.34	21.49
	3.0	2.46	82.00	3.66	5.78
	5.0	3.59	71.80	11.42	14.09
Taleranol	1.0	0.71	71.0	15.49	20.87
	3.0	2.41	80.34	6.23	12.17
	5.0	4.48	89.60	12.73	16.43
Amoxicillin	25.0	15.47	61.89	9.63	19.45
	50.0	30.64	61.28	2.34	16.99
	75.0	59.63	79.50	9.11	10.48
Oxacillin	150.0	159.36	106.22	8.78	15.16
	300.0	278.48	92.83	10.49	15.86
	450.0	423.35	94.08	9.71	14.20
Cloxacallin	25.0	19.74	78.96	13.98	20.71
	50.0	37.47	74.94	9.45	14.19
	75.0	64.78	86.37	11.07	13.71
Benzylpenicillin	25.0	21.35	85.44	15.04	16.84
	50.0	51.28	102.56	5.44	12.49
	75.0	71.54	95.39	5.90	8.11
Ampicillin	25.0	18.91	75.64	6.51	17.10
	50.0	41.45	82.90	4.80	7.16
	75.0	72.0	96.00	16.83	19.35
Ceftioflur	500.0	478.22	95.64	3.59	4.40
	1000.0	923.18	92.32	1.87	2.89
	1500.0	1399.28	93.29	1.80	2.30
Cephalexin	100.0	105.18	105.18	9.05	12.66
	200.0	213.32	106.66	3.11	4.70
	300.0	288.35	96.12	1.81	2.30
Enrofloxacin	50.0	56.89	113.78	2.26	12.44
	100.0	76.71	76.71	8.90	16.95
	150.0	123.36	82.24	7.52	11.98
Ciprofloxacin	50.0	40.56	81.13	25.88	34.04
	100.0	83.35	83.35	8.72	8.86
	150.0	139.22	92.81	1.84	10.61
Oxytetracycline	50.0	39.54	79.08	6.53	10.13
	100.0	77.25	77.25	4.85	8.65
	150.0	128.35	85.56	5.55	8.41
Sulfachloropyridazine	50.0	53.64	107.27	9.29	19.39
	100.0	82.92	82.92	6.60	10.97
	150.0	142.68	95.12	2.28	6.84
Sulfadiazine	50.0	48.49	96.98	8.60	16.12
	100.0	74.94	74.94	6.60	7.08
	150.0	135.92	90.61	6.71	8.04
Sulfadimetoxine	50.0	37.36	74.72	4.70	9.28
	100.0	85.66	85.66	17.19	18.72
	150.0	141.56	94.37	4.34	9.28
Sulfadimidine	50.0	43.15	86.30	2.11	5.22
	100.0	96.57	96.57	4.89	9.15
	150.0	148.84	99.23	5.23	8.60
Sulfamethoxazol	50.0	50.63	101.26	5.58	5.86
	100.0	84.19	84.19	4.42	7.37
	150.0	138.52	92.35	2.73	4.87
Carbofuran	5.0	3.99	79.8	5.26	10.37
	10.0	9.25	92.52	15.78	19.75
	15.0	14.54	96.93	8.62	9.78
Carbaryl	25.0	21.51	86.04	3.99	14.07
	50.0	51.10	102.20	15.38	19.39
	75.0	77.23	102.97	6.75	10.92
Parathion	25.0	16.32	65.28	7.54	9.27
	50.0	36.18	72.36	5.84	7.93
	75.0	59.87	79.82	8.37	10.23
Malathion	10.0	7.45	74.51	3.22	9.50
	20.0	17.38	86.9	7.02	7.57
	30.0	26.17	87.22	3.52	7.68
Diazinon	10.0	9.21	92.14	9.12	13.07
	20.0	19.93	99.65	6.14	10.75
	30.0	28.11	93.70	1.71	8.64
Dimethoate	25.0	16.46	65.84	4.01	5.86
	50.0	35.78	71.56	9.17	18.61
	75.0	59.85	79.80	7.45	7.61
Atrazine	25.0	21.45	85.80	8.96	10.29
	50.0	49.78	99.56	6.45	9.47
	75.0	69.64	92.85	2.75	5.66
Permethrin	25.0	26.13	104.52	11.35	13.43
	50.0	51.48	102.96	7.81	12.45
	75.0	78.13	104.17	0.97	2.85
Cypermethrin	1000.0	996.48	99.65	4.23	5.84
	2000.0	1848.52	92.43	2.30	3.11
	3000.0	2921.66	97.39	1.54	2.27
Deltamethrin	15.0	12.66	84.40	2.21	4.81
	30.0	27.38	92.60	4.49	14.27
	45.0	39.12	86.93	15.39	18.62
Coumaphos	10.0	8.49	84.90	10.83	16.42
	20.0	17.86	89.30	11.25	16.04
	30.0	28.33	94.43	8.75	14.70
Dichlorophos	25.0	23.84	95.36	1.22	2.33
	50.0	47.56	95.15	4.65	5.42
	75.0	73.12	97.49	3.47	6.69
Chlorpyrifos	5.0	3.28	65.6	2.26	6.43
	10.0	7.11	71.15	1.54	2.78
	15.0	11.64	77.60	8.51	12.64
Fenvalerate	12.5	9.22	61.47	4.99	11.62
	25.0	20.35	81.4	3.56	7.58
	50.0	35.18	70.36	14.84	19.64
Zearalenone	25.0	20.46	81.84	11.15	13.38
	50.0	42.18	84.36	4.89	9.97
	75.0	69.88	93.07	6.02	9.50
Ochratoxin A	25.0	18.46	73.84	5.15	6.89
	50.0	33.58	67.15	4.11	6.00
	75.0	65.14	86.85	11.02	13.86

Statistical evaluation revealed that average recoveries were in the range of 61.28 (amoxicillin at 50 μg/kg) to 120% (terbutaline at 0.5 μg/kg) and CV for the intra-day precision were in the range of 0.97 (for permethrin at 75 μg/kg) to 25.93% (isoxsuprin at 0.2 μg/kg). For inter-day precision the CV was from 2.27% (for cypermetrine at 3000 μg/kg) to 34.04% (for ciprofloxacin at 50 μg/kg). For all investigated compounds, the average recoveries and CV for precision was acceptable and in agreement with the criteria of Commission Decision 2002/657/EC and SANTE/12682/2019. To further demonstrate the suitability of the method the laboratory will be participate in proficiency tests in the near future.

#### Real sample analysis

A total of 87 import and local samples from bovine meat were analysed, in order to test the applicability of the established method for the routine analysis. After the sample analysis the trace residues of enrofloxacin, oxytetracycline and sulfadiazine at the concentration of 35.22 µg/kg, 27.35 µg/kg, and 36.20 µg/kg, respectively, were confirmed in three samples. The chromatograms from positive samples are given in Additional file 2: Figs. S1–S3.

## Conclusions

A multi-class and multi-residue analytical methods including use of stable isotopes were developed for analysis of residues of veterinary drugs and other contaminants in bovine meat. The veterinary drugs included antimicrobials (β-lactams, fluoroquinolones, cephalosporins, tetracyclines and sulphonamides), anabolic hormones, lactones, β-agonists and from pesticides and mycotoxins (zearalenone and ochratoxin A). Thirteen (13) stable isotopes were used as internal standards to cover wide range of analytes including veterinary drug residues, pesticides and mycotoxins. The developed method showed good performance characteristics of the method comply with EU recommendations.

## Supplementary Information

**Additional file 1.** Recoveries from investigation of sample amount and extraction steps.

**Additional file 2.** Chromatograms of positive samples from bovine meat.

## Data Availability

All data and materials analyzed during the current study are included in this manuscript.

## Abbreviations

CCα: Decision limit
CCβ: Detection capability
LOD: Limit of detection
LOQ: Limit of quantification
CV: Coefficient of variation
LC–MS/MS: Liquid chromatography tandem mass spectrometry
SPE: Solid-phase extraction
QuEChERS: Quick, easy, cheap, effective, rugged, and safe
MeCN: Acetronitrile
MeOH: Methanol
MRL: Maximum recommended residue levels
MRPL: Minimum required performance limits
EtOAc: Ethylacetate
MRM: Multiple reaction monitoring
CRMs: Certified reference materials
ESI: Electrospray ionisation
S/N: Signal to noise ratio
R^2^: Coefficient of corelation

## References

[1] Anastassiades M, Lehotay SJ, Stajnbaher D, Schenk FJ (2003). Fast and easy multiresidue method employing acetonitrile extraction/partitioning and “dispersive solid-phase extraction” for the determination of pesticide residues in produce. J AOAC Int.

[2] Aguilera-Luiz MM, Martínez Vidal JL, Romero-González R, Garrido FA (2008). Multiresidue determination of veterinary drugs in milk by ultra-high pressure liquid chromatography tandem mass spectrometry. J Chromatogr A.

[3] Biselli S, Schwalb U, Meyer A, Hartig L (2013). A multi-class, multi-analyte method for routine analysis of 84 veterinary drugs in chicken muscle using simple extraction and LC-MS/MS. Food Addit Contam Part A.

[4] Bitas D, Kabir A, Locatelli M, Samanidou V (2018). Food sample preparation for the determination of sulfonamides by high-performance liquid chromatography: state-of-the-art. Separations.

[5] Danezis GP, Anagnostopoulos CJ, Liapis K, Koupparis MA (2016). Multi-residue analysis of pesticides, plant hormones, veterinary drugs and mycotoxins using HILIC chromatography—MS/MS in various food matrices. Anal Chim Acta.

[6] European Commission. Decision (2002/657/EC) of 12 August 2002 implementing Council Directive 96/23/EC concerning the performance of analytical methods and interpretation of results. OJEU, L221*,* 2002. 8–36.

[7] European Commision. Commision Reguation (EU) No. 1881/2006 of 19 December 2006: setting maximum levels for certain contaminants in foodstuffs. OJEU*,* L364/5, 2006. 1–20.

[8] European Commission. Commission Regulation (EU) No. 37/2010 of 22 December 2009: on pharmacologically active substances and their classification regarding maximum residue limits in foodstuffs of animal origin. OJEU, L15, 2010. 1–72.

[9] European Commission. SANTE/12682/2019. Guidance document on analytical quality control and method validation procedures for pesticide residues analysis in food and feed. 2019.

[10] EU pesticide database. https://ec.europa.eu/food/plant/pesticides/eu-pesticides-database/public/?event=pesticide.residue.selection&language=EN.

[11] Gómez-Pérez ML, Romero-González R, Vidal JLM, Frenich AG (2015). Analysis of pesticide and veterinary drug residues in baby food by liquid chromatography coupled to Orbitrap high resolution mass spectrometry. Talanta.

[12] Kaufmann A, Butcher P, Maden K, Widmer M (2008). Quantitative multiresidue method for about 100 veterinary drugs in different meat matrices by sub 2-μm particulate high-performance liquid chromatography coupled to time of flight mass spectrometry. J Chromatogr A.

[13] Marazuela MD, Fanali S, Haddad PR, Poole C, Riekkola ML (2017). Determination of veterinary drug residues in foods by liquid chromatography-mass spectrometry: basic and cutting-edge applications. Liquid chromatography: applications.

[14] Reig M, Toldrá F (2008). Veterinary drug residues in meat: concerns and rapid methods for detection. Meat Sci.

[15] Ren Y, Zhang Y, Shao S, Cai Z, Feng L, Pan H, Wang Z (2007). Simultaneous determination of multi-component mycotoxin contaminants in foods and feeds by ultra-performance liquid chromatography–tandem mass spectrometry. J Chromatogr A.

[16] SANCO. CRLs view on state of the art analytical methods for national residue control plans. CRL Guidance Paper (7 December 2007) 2007. pp. 1–8.

[17] Stubbings G, Bigwood T (2009). The development and validation of a multiclass liquid chromatography tandem mass spectrometry procedure for the determination of veterinary drug residues in animal tissue using a QuEChERS (QUick, Easy, CHeap, Effective, Rugged and Safe) approach. Anal Chim Acta.

[18] Villar-Pulido M, Gilbert-López B, García-Reyes JF, Ramos-Martos N, Molina-Díaz A (2011). Multiclass detection and quantitation of antibiotics and veterinary drugs in shrimps by fast liquid chromatography time-of-flight mass spectrometry. Talanta.

[19] Wei H, Tao Y, Chen D, Xie S, Pan Y, Liu Z, Huang L, Yuan Z (2015). Development and validation of a multi-residue screening method for veterinary drugs, their metabolites and pesticides in meat using liquid chromatography-tandem mass spectrometry. Food Addit Contam Part A.

[20] Whelan M, Kinsella B, Furey A, Moloney M, Cantwell H, Letohay SJ, Danaher M (2010). Determination of anthelmintic drug residues in milk using ultra high-performance liquid chromatography-tandem mass spectrometry with rapid polarity switching. J Chromatogr A.

[21] Xie J, Peng T, Zhu A, He J, Chang Q, Hu X, Chen H, Fan C, Jiang W, Chen M, Li J, Ding S, Jiang H (2015). Multi-residue analysis of veterinary drugs, pesticides and mycotoxinsin dairy products by liquid chromatography–tandem mass spectrometry using low-temperature cleanup and solid phase extraction. J Chromatogr B Analyt Technol Biomed Life Sci.

[22] Yamada R, Kozono M, Ohmori T, Morimatsu F, Kitayama M (2006). Simultaneous determination of residual veterinary drugs in bovine, porcine, and chicken muscle using liquid chromatography coupled with electrospray ionization tandem mass spectrometry. Biosci Biotechnol Biochem.

[23] Zhan J, Xu DM, Wang SJ, Sun J, Xu YJ, Ni ML, Yin JY, Chen J, Yu XJ, Huang ZQ (2013). Comprehensive screening for multi-class veterinary drug residues andother contaminants in muscle using column-switching UPLC–MS/MS. Food Addit Contam Part A.

[24] Zhan J, Zhong YY, Yu XJ, Peng JF, Chen SB, Yin JY, Zhang JJ, Zhu Y (2013). Multi-class method 24 for determination of veterinary drug residues and other contaminants in infant formula by ultra performance liquid chromatographytandem mass spectrometry. Food Chem.

