# Supplementary material for: A new LC–MS/MS method for multiple residues/contaminants in bovine meat
Source: BMC Chem. 2021 Dec 8;15(1):62. doi: 10.1186/s13065-021-00788-5 (PMC8656019; doi:10.1186/s13065-021-00788-5)
Supplement: Supplementary file 1 — Additional file 1. Recoveries from investigation of sample amount and extraction steps. [file 13065_2021_788_MOESM1_ESM.docx]

Additional files. Table S1. Recoveries from investigation of sample amount and extraction steps

|  |  | Recovery (%) | | | |
| --- | --- | --- | --- | --- | --- |
| Analytes | Added concentration  (µg/kg) | Mass of samples – 5 g | | Mass of samples – 10 g | |
|  |  | Extraction  1x20 ml | Extraction  2x20 ml | Extraction  1x20 ml | Extraction  2x20 ml |
| Clenbuterol | 0.05 | 67.35 | 75.28 | 98.25 | 104.00 |
|  | 0.25 | 62.11 | 79.45 | 93.24 | 105.20 |
|  | 0.50 | 71.12 | 81.46 | 89.78 | 102.20 |
| Brombuterol | 0.05 | 54.35 | 64.13 | 70.46 | 75.64 |
|  | 0.25 | 61.34 | 64.11 | 65.12 | 92.01 |
|  | 0.50 | 51.22 | 60.08 | 72.84 | 95.34 |
| Mabuterol | 0.05 | 59.37 | 70.00 | 72.14 | 76.00 |
|  | 0.25 | 57.14 | 64.12 | 81.46 | 92.00 |
|  | 0.50 | 51.34 | 61.78 | 75.15 | 95.40 |
| Clenpenterol | 0.2 | 61.45 | 70.01 | 80.12 | 87.50 |
|  | 0.5 | 60.12 | 72.14 | 75.46 | 93.80 |
|  | 0.75 | 64.17 | 67.51 | 77.12 | 95.47 |
| Isoxsuprin | 0.2 | 54.78 | 70.05 | 80.14 | 88.50 |
|  | 0.5 | 61.13 | 64.32 | 82.15 | 106.40 |
|  | 0.75 | 67.48 | 74.13 | 94.12 | 102.00 |
| Cimbuterol | 0.2 | 47.78 | 59.78 | 69.17 | 95.47 |
|  | 0.5 | 52.36 | 63.14 | 71.31 | 99.40 |
|  | 0.75 | 49.12 | 57.15 | 62.15 | 94.53 |
| Ractopamine | 0.2 | 47.78 | 63.14 | 75.12 | 96.50 |
|  | 0.5 | 43.12 | 65.78 | 88.14 | 104.40 |
|  | 0.75 | 51.36 | 59.74 | 80.22 | 102.93 |
| Salbutamol | 0.5 | 44.16 | 60.36 | 71.36 | 108.00 |
|  | 0.75 | 49.78 | 55.14 | 67.34 | 94.93 |
|  | 1.0 | 41.36 | 57.78 | 75.44 | 86.60 |
| Zilpaterol | 0.5 | 54.66 | 67.78 | 75.12 | 81.40 |
|  | 0.75 | 59.71 | 61.46 | 77.36 | 98.93 |
|  | 1.0 | 63.17 | 70.08 | 80.12 | 91.10 |
| Terbutaline | 0.5 | 49.51 | 62.46 | 71.46 | 116.20 |
|  | 0.75 | 50.12 | 65.71 | 67.35 | 93.20 |
|  | 1.0 | 59.78 | 60.14 | 71.14 | 77.40 |
| Testosterone | 1.0 | 48.78 | 60.02 | 67.46 | 71.50 |
|  | 3.0 | 52.14 | 57.46 | 69.14 | 71.66 |
|  | 5.0 | 56.78 | 61.12 | 63.18 | 71.60 |
| Boldenone | 1.0 | 64.36 | 71.14 | 80.04 | 92.0 |
|  | 3.0 | 60.08 | 73.15 | 79.12 | 81.34 |
|  | 5.0 | 69.35 | 73.96 | 74.56 | 77.80 |
| Clostebol | 1.0 | 68.78 | 69.36 | 90.14 | 104.0 |
|  | 3.0 | 60.14 | 75.14 | 79.36 | 109.34 |
|  | 5.0 | 71.35 | 80.24 | 85.14 | 100.80 |
| Methyltestosterone | 1.0 | 44.66 | 57.78 | 64.36 | 80.0 |
|  | 3.0 | 49.51 | 60.06 | 67.78 | 72.67 |
|  | 5.0 | 42.78 | 51.35 | 73.14 | 90.20 |
| Stanozolol | 1.0 | 67.35 | 70.12 | 80.04 | 93.0 |
|  | 3.0 | 71.14 | 78.35 | 81.14 | 91.33 |
|  | 5.0 | 70.02 | 71.32 | 74.35 | 89.20 |
| 19 nortestosterone | 1.0 | 70.02 | 71.45 | 95.14 | 105.0 |
|  | 3.0 | 64.13 | 79.35 | 83.11 | 113.67 |
|  | 5.0 | 67.78 | 92.14 | 97.78 | 104.20 |
| Zeranol | 1.0 | 47.78 | 52.14 | 62.11 | 75.0 |
|  | 3.0 | 46.42 | 50.00 | 67.78 | 82.00 |
|  | 5.0 | 43.56 | 55.64 | 69.75 | 71.80 |
| Taleranol | 1.0 | 57.78 | 64.35 | 67.78 | 71.0 |
|  | 3.0 | 65.14 | 70.12 | 64.35 | 80.34 |
|  | 5.0 | 61.11 | 67.78 | 74.38 | 89.60 |
| Amoxicillin | 25.0 | 50.04 | 53.15 | 59.48 | 61.89 |
|  | 50.0 | 47.35 | 57.78 | 61.00 | 61.28 |
|  | 75.0 | 41.35 | 49.12 | 57.14 | 79.50 |
| Oxacillin | 150.0 | 70.00 | 75.36 | 84.35 | 106.22 |
|  | 300.0 | 64.35 | 79.14 | 80.12 | 92.83 |
|  | 450.0 | 69.04 | 70.12 | 91.13 | 94.08 |
| Cloxacallin | 25.0 | 57.35 | 60.25 | 73.14 | 78.96 |
|  | 50.0 | 54.15 | 67.48 | 70.04 | 74.94 |
|  | 75.0 | 60.80 | 61.35 | 74.35 | 86.37 |
| Benzylpenicillin | 25.0 | 47.45 | 63.12 | 80.12 | 85.44 |
|  | 50.0 | 42.11 | 61.78 | 85.45 | 102.56 |
|  | 75.0 | 51.46 | 65.17 | 91.13 | 95.39 |
| Ampicillin | 25.0 | 45.78 | 60.08 | 63.17 | 75.64 |
|  | 50.0 | 51.12 | 57.14 | 75.23 | 82.90 |
|  | 75.0 | 42.46 | 62.42 | 67.78 | 96.00 |
| Ceftioflur | 500.0 | 73.18 | 87.14 | 92.11 | 95.64 |
|  | 1000.0 | 71.56 | 80.22 | 90.46 | 92.32 |
|  | 1500.0 | 79.37 | 85.14 | 91.35 | 93.29 |
| Cephalexin | 100.0 | 85.46 | 90.15 | 96.11 | 105.18 |
|  | 200.0 | 80.04 | 91.35 | 102.11 | 106.66 |
|  | 300.0 | 83.14 | 94..00 | 94.13 | 96.12 |
| Enrofloxacin | 50.0 | 61.46 | 72.11 | 77.46 | 113.78 |
|  | 100.0 | 57.75 | 60.08 | 70.02 | 76.71 |
|  | 150.0 | 59.72 | 66.42 | 69.46 | 82.24 |
| Ciprofloxacin | 50.0 | 60.02 | 67.42 | 77.46 | 81.13 |
|  | 100.0 | 57.71 | 61.35 | 70.02 | 83.35 |
|  | 150.0 | 52.15 | 66.15 | 74.13 | 92.81 |
| Oxytetracycline | 50.0 | 47.78 | 57.52 | 62.11 | 79.08 |
|  | 100.0 | 45.36 | 50.04 | 60.08 | 77.25 |
|  | 150.0 | 52.13 | 61.32 | 67.78 | 85.56 |
| Sulfachloropyridazine | 50.0 | 60.08 | 75.14 | 85.46 | 107.27 |
|  | 100.0 | 65.35 | 70.06 | 85.55 | 82.92 |
|  | 150.0 | 59.78 | 73.15 | 79.32 | 95.12 |
| Sulfadiazine | 50.0 | 51.35 | 62.25 | 80.46 | 96.98 |
|  | 100.0 | 50.08 | 60.08 | 77.46 | 74.94 |
|  | 150.0 | 56.35 | 71.31 | 74.11 | 90.61 |
| Sulfadimetoxine | 50.0 | 47.78 | 63.46 | 67.78 | 74.72 |
|  | 100.0 | 45.12 | 65.15 | 61.35 | 85.66 |
|  | 150.0 | 51.35 | 57.76 | 60.12 | 94.37 |
| Sulfadimidine | 50.0 | 54.78 | 62.14 | 75.15 | 86.30 |
|  | 100.0 | 62.71 | 72.80 | 78.78 | 96.57 |
|  | 150.0 | 66.75 | 69.35 | 71.35 | 99.23 |
| Sulfamethoxazol | 50.0 | 57.78 | 71.35 | 80.08 | 101.26 |
|  | 100.0 | 63.12 | 79.88 | 84.00 | 84.19 |
|  | 150.0 | 67.75 | 74.33 | 75.35 | 92.35 |
| Carbofuran | 5.0 | 60.12 | 65.15 | 74.35 | 79.8 |
|  | 10.0 | 57.53 | 62.23 | 77.78 | 92.52 |
|  | 15.0 | 55.14 | 60.14 | 80.12 | 96.93 |
| Carbaryl | 25.0 | 71.35 | 80.45 | 89.35 | 86.04 |
|  | 50.0 | 77.14 | 82.14 | 88.14 | 102.20 |
|  | 75.0 | 70.08 | 80.15 | 95.46 | 102.97 |
| Parathion | 25.0 | 44.14 | 49.56 | 60.07 | 65.28 |
|  | 50.0 | 40.08 | 53.58 | 63.14 | 72.36 |
|  | 75.0 | 47.78 | 55.14 | 59.78 | 79.82 |
| Malathion | 10.0 | 52.11 | 50.04 | 60.45 | 74.51 |
|  | 20.0 | 47.46 | 53.55 | 59.78 | 86.9 |
|  | 30.0 | 52.14 | 55.14 | 59.17 | 87.22 |
| Diazinon | 10.0 | 69.25 | 78.18 | 90.12 | 92.14 |
|  | 20.0 | 71.35 | 72.14 | 91.46 | 99.65 |
|  | 30.0 | 68.80 | 77.46 | 87.78 | 93.70 |
| Dimethoate | 25.0 | 42.14 | 47.78 | 54.13 | 65.84 |
|  | 50.0 | 47.78 | 49.51 | 59.46 | 71.56 |
|  | 75.0 | 40.08 | 52.14 | 57.14 | 79.80 |
| Atrazine | 25.0 | 57.78 | 62.11 | 74.13 | 85.80 |
|  | 50.0 | 52.15 | 60.54 | 77.46 | 99.56 |
|  | 75.0 | 54.35 | 69.17 | 85.14 | 92.85 |
| Permethrin | 25.0 | 71.35 | 88.15 | 92.51 | 104.52 |
|  | 50.0 | 64.13 | 80.15 | 87.56 | 102.96 |
|  | 75.0 | 72.48 | 86.46 | 93.14 | 104.17 |
| Cypermethrin | 1000.0 | 80.00 | 88.46 | 95.14 | 99.65 |
|  | 2000.0 | 85.28 | 89.12 | 90.08 | 92.43 |
|  | 3000.0 | 83.17 | 83.48 | 94.35 | 97.39 |
| Deltamethrin | 15.0 | 47.78 | 57.17 | 65.22 | 84.40 |
|  | 30.0 | 50.02 | 51.31 | 60.08 | 92.60 |
|  | 45.0 | 50.07 | 53.15 | 61.31 | 86.93 |
| Coumaphos | 10.0 | 42.78 | 53.54 | 63.14 | 84.90 |
|  | 20.0 | 47.13 | 50.11 | 65.77 | 89.30 |
|  | 30.0 | 41.11 | 55.45 | 60.09 | 94.43 |
| Dichlorophos | 25.0 | 75.35 | 80.54 | 90.08 | 95.36 |
|  | 50.0 | 71.08 | 85.12 | 87.56 | 95.15 |
|  | 75.0 | 80.00 | 81.34 | 80.02 | 97.49 |
| Chlorpyrifos | 5.0 | 41.35 | 52.08 | 60.08 | 65.6 |
|  | 10.0 | 45.12 | 47.35 | 54.35 | 71.15 |
|  | 15.0 | 42.11 | 50.08 | 65.14 | 77.60 |
| Fenvalerate | 12.5 | 42.11 | 47.78 | 57.75 | 61.47 |
|  | 25.0 | 40.08 | 52.11 | 67.17 | 81.4 |
|  | 50.0 | 45.46 | 49.43 | 66.08 | 70.36 |
| Zearalenone | 25.0 | 51.57 | 66.36 | 75.36 | 81.84 |
|  | 50.0 | 59.75 | 61.17 | 70.08 | 84.36 |
|  | 75.0 | 55.14 | 69.35 | 79.35 | 93.07 |
| Ochratoxin A | 25.0 | 41.35 | 50.08 | 60.18 | 73.84 |
|  | 50.0 | 44.13 | 53.55 | 62.35 | 67.15 |
|  | 75.0 | 40.08 | 59.17 | 61.48 | 86.85 |
