# Supplementary material for: A new LC–MS/MS method for multiple residues/contaminants in bovine meat
Source: BMC Chem. 2021 Dec 8;15(1):62. doi: 10.1186/s13065-021-00788-5 (PMC8656019; doi:10.1186/s13065-021-00788-5)

Additional files

Figure S1. Chromatogram of positive sample from bovine meat - Enrofloxacin


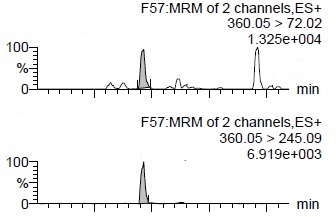


Figure S2. Chromatogram of positive sample from bovine meat – Oxytetracycline


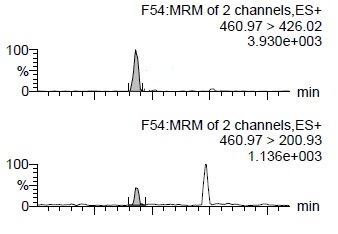


Figure S3. Chromatogram of positive sample from bovine meat – Sulfadiazine


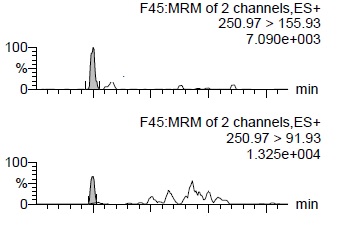

Supplement: Supplementary file 2 — Additional file 2. Chromatograms of positive samples from bovine meat. [file 13065_2021_788_MOESM2_ESM.docx]
